# Inflammatory cytokines via up-regulation of aquaporins deteriorated the pathogenesis of early osteoarthritis

**DOI:** 10.1371/journal.pone.0220846

**Published:** 2019-08-12

**Authors:** Chunjiang Tan, Jiahui Zhang, Wenlie Chen, Fangfang Feng, Chao Yu, Xiaodong Lu, Ruhui Lin, Zuanfang Li, Yunmei Huang, Liangpu Zheng, Meiya Huang, Guangwen Wu

**Affiliations:** 1 Fujian University of Traditional Chinese Medicine, Fuzhou, Fujian, China; 2 Fujian Academy of Integrative Medicine, Fuzhou, Fujian, China; 3 Fujian Key Laboratory of Integrative Medicine on Geriatrics, Fuzhou, Fujian, China; 4 The First Hospital of Fuzhou, Fuzhou, Fujian, China; 5 National Laboratory of Traditional Chinese Medicine on Pharmacology (Cell Structure and Function), Fuzhou, Fujian, China; Universite de Nantes, FRANCE

## Abstract

**Background:**

Inflammatory cytokines enhanced the progress of the pathogenesis of osteoarthritis, however the mechanisms remain unclear. The objective is to determine aquaporins (AQPs) in the pathogenesis of osteoarthritis.

**Methods and findings:**

Primary rat articular chondrocytes were treated with IL-1β to mimic the early stage of osteoarthritis in vitro. Early osteoarthritis animal model was established by intra-articular injection of 4% papain. Micro- or ultra-structure histopathologic changes, cell viability, apoptosis cells and cell membrane permeability, locations and expressions of AQP1 and AQP3 and matrix were detected in the cartilage or in the chondrocytes of knee. IL-1β could reduce the chondrocytes viability, increase the apoptosis cells, and also impair the cell membrane and organelles. IL-1β significantly induced the up-regulation of AQP1 and AQP3 in the chondrocytes. In the chondrocytes, AQPs were mainly clustered in both membrane and perinuclear region of cytoplasm, while higher AQPs were detected in the superficial and middle layers of the cartilage. With the up-regulation of AQPs, the cartilage matrix was considerably decreased in both the chondrocytes and in the osteoarthritis cartilage. In the early osteoarthritis rat model, serum and synovial fluid confirmed that higher IL-1β could increase the expressions of AQPs, and decrease the cartilage matrix in both the chondrocytes and the cartilage.

**Conclusions:**

Inflammatory cytokine IL-1β via up-regulation of AQPs caused the abnormal metabolism of water transport and loss of the cartilage matrix in the chondrocytes, and ultimately exacerbated the pathogenesis of early osteoarthritis. Therefore, AQPs may be a candidate therapeutic target for prevention and treatment of osteoarthritis.

## Introduction

Osteoarthritis (OA) is a degenerative joint disease that mainly involves cartilage injury.[1, 2] Water, consisting of over 70% of the total cartilage weight, is divided into bound water and free water. The bound water plays a vital role in shock absorption, load transmission and stability. Free water nourishes and lubricates the articular cartilage jointly with the synovial fluid. [3, 4] Therefore, the total water content in cartilage, or the alterations in the proportion of bound water and free water has great influence on OA.

Aquaporins (AQPs), a family consisted of more than ten different membrane protein channels, play a critical role in controlling the water content of cells. [5] Recent research suggested that the high AQP1 mRNA was detected in the cartilage in the OA rat model, [6] or in the chondrocytes from osteoarthritis knee menisci. [7] Similar high levels of AQP3 mRNA and protein were found in different parts of OA cartilage, [8] whose early stage is characterized by swelling with increased water content. [9] Thus, AQP1 and AQP3 may act as additional candidates for cartilage damage in OA. [10, 11]

Chondrocytes apoptosis is part of the important mechanisms of articular cartilage degeneration in OA pathogenesis.[2, 12] Proinflammatory cytokines as IL-1β and tumor necrosis factor α (TNF-α), via triggering apoptosis of chondrocytes, played a key role in the pathogenesis of osteoarthritis articular, [13, 14, 15] which in turn increased the inflammatory mediators. [16] In addition, these cytokines could activate a variety of extracellular matrix degrading enzymes, inhibiting collagen II and proteoglycan synthesis, which resulted in alterations in cartilage matrix components and the damage of cartilage structure and functions. [17, 18] However, it has not been determined whether these inflammatory factors via AQPs participate in the pathogenesis of OA.

We hypothesized that inflammatory cytokines, via AQPs altering the water content in the cartilage matrix, participated in the pathogenesis of OA. In this study, we established the cell and OA animal model, the pathological changes, the expressions of AQPs, and water content in chondrocytes and articular cartilage were detected, trying to elucidate the role of AQPs in the pathogenesis of OA.

## Materials and methods

### Isolation, culture and identification of chondrocytes

Rat articular chondrocytes were isolated as before, [19] and cultured under standard conditions (5% CO_2_, 95% air) in sterile Dulbecco's modified Eagle medium supplemented with 10% fetal bovine serum. The second generation cells were identified and confirmed with type II collagen by immunocytochemistry. Images of chondrocytes were captured by phase-contrast microscope (Olympus, Japan).

### Chondrocytes MTT assay

The cells were seeded in 96-well plates (100μl/well) with the density of 5×10^4^cells/ml. After incubation for 24h, the cells were incubated with or without IL-1β (10ng/ml) respectively for 24h to mimic OA cell model. [20, 21] Then, the cells were incubated with 1 mg/ml 3-(4,5-dimethyl-2-thiazolyl)-2,5-diphenyl-2H tetrazolium bromide (MTT, Sigma, USA) for 4h. The supernatant was replaced by 150ul dimethylsulfoxide (DMSO) for 10 min then measured at 570 nm absorbance (Exl 800, Biotek Instruments, USA).

### Quantitative determination of apoptosis cells

After treatment with IL-1β, the rate of apoptosis cells was determined by flow cytometric analysis using a fluorescence-activated cell sorting (FACS Caliber, Becton-Dickinson, USA) and the Annexin V/Pi apoptosis dection kit (San Jose, USA).

### Cell membrane permeability tracing

After pre-fixed in 3% glutaraldehyde -1% Lanthanum nitrate -0.1mol/L sodium dimethylarsenate (pH7.4) for 48h, the cells were fixed with 1% osmium in 0.1mol/L sodium dimethylarsenate for 2h and dehydrated in ethanol solutions and acetone, followed by embedding in Epon 618. The samples were sectioned to 90nm without staining. The ultrathin sections were observed and images were captured under TEM (H 7650, HITACHI, Japan) at 80KV.

### Proteoglycan content evaluations

Toluidine blue staining was used to assess the content of proteoglycan in chondrocytes. Firstly, the slips were washed twice with PBS, fixed for 0.5 h in 4% paraformaldehyde at 4℃ followed by staining with 0.1% Toluidine blue for 20min in RT. Then the slides were washed twice with PBS and observed and photographed under the microscope (MDL, Leica, Germany). Semiquantitative analysis was conducted by medical image analysis system (Motic Med 6.0, China).

### AQPs and collagen II semi-quantitative analysis

The treated cells were fixed in 4% paraformaldehyde at 4℃ for 0.5 h followed by treating with 0.1% Triton in PBS for 10min, blocking with 5% normal goat serum and 10% bovine serum albumin. Then the cells were incubated with antibody (AQP1, 1:200 dilution; AQP3, 1:300 dilution, Collagen II 1:200 dilution) overnight at 4˚C. The cells were incubated with secondary goat anti-rabbit antibody for 2h RT and reacted with DAB for 10min before treated with hematoxylin for 2 min. The images of slides were captured and semi-quantitative analysis was performed using Image Pro-Plus 6.0 (Media cybernetics, Inc.)

### AQPs immunofluorescence location

Immunolcytochemistry as described before, the cell slides were incubated with the primary antibody (AQP1, 1:50 dilution; AQP3, 1:100 dilution) or the secondary goat anti-rabbit fluorescently labeled antibody for 2h RT, respectively. After washing with PBS, the immunofluorescences as the location of AQPs proteins were observed under a laser scanning confocal microscope (LSM 710, Zeiss, Germany).

### Establishment of early OA animal model and preparation of samples

A total of 16 Sprague-Dawley rats, weighing 200±10g, (Male, 6 weeks old, from Shanghai SLAC Laboratory Animal Co., Ltd.) were randomly divided equally into OA and control groups, and habituated to the laboratory environment for at least 7 days before experimentation. On the day of the experiment, the rats were anaesthetized with chloralhydrate (0.4 g/kg) i.p. After being anesthetized, the rats skin overlying the left knees was shaved and a 2 cm longitudinal skin incision exposing the knee,0.2ml papain solution was injected intra-articularly in the left knee joint to induce early OA model, continuing 3 times and 3 days interval in each as before, [22] another 8 rats served as the control group.

All rats were maintained under identical conditions which included temperature of 24℃, 60% relative humidity, and food and water ad libitum. Synovial fluid samples were obtained from rat joints as described before.[23] Two needles joined together were inserted into the knee joint of anaesthetised rats and connected to a perfusion pump (Watson-Marlow Fluid Technology Group, model: 101U). Sterile saline was infused and withdrawn at 100 μl min^-1^ until a 250 μl sample was collected.Synovial fluid samples, rapidly cooled and stored at -45 °C until analysis, were obtained from rat joints After animals sacrificed, the left knee joint samples were obtained for further examination.

The histopathological changes scored 3–10 assessed by Mankin’s grading system were regarded the successful OA model. The care and use of the animals in the present study were approved and formally abided by the Animal Care and Use Committee of Fujian University of Traditional Chinese Medicine.

### Determination of AQPs and collagen II by immunohistochemistry

The sections were kept in sodium citrate solution for antigen retrieval. After that, the sections were incubated for 10 min in 0.3% H_2_O_2_, followed by blocking in non-specific antibody binding by PBS containing 10% goat serum. The sections were incubated with a rabbit polyclonal antibody AQP1 (1:600 dilution) or AQP3 (1:400 dilution) or Collagen II (1:500 dilution) overnight at 4℃, respectively. After washing 3 times for 5 min each in PBS, the sections were incubated with horseradish peroxidase labeled polymer conjugated to a goat anti-rabbit immunoglobulins for 20 min at RT. DAB chromogen were utilized to visualize antibody labeling. Sections were counterstained with hematoxylin. Then images were captured and semi-quantitative analysis was performed using medical image analysis system (Motic Med 6.0, China).

### AQPs analysis by laser scanning confocal microscopy

The sections were blocked with 10% goat serum in PBS for 1h, followed by labeling with an antibody (AQP1, 1:200; AQP3, 1:40) overnight at 4℃. All sections were incubated with an Alexa FluorTM 488 (dilution 1:600) for 2h at RT, and then incubated with 4',6-Diamidino-2-phenylindole (DAPI) for 5min and washed with PBS. After mounted in anti-quenching mounting medium, the sections were observed and photographed under LSCM (LSM 710, Zeiss, Germany).

### Inflammatory factors detection by radioimmunoassay

IL-1β in serum and synovial fluid were measured by radioimmunoassay according to the procedures in the kit (125I-IL-1β, North Biotechnology research institute, China).

### Statistical analysis

All data were represented as mean ± standard deviation and analyzed by using the SPSS 20.0. Differences between two groups were determined using the Student’s t-test or non-parametric Mann-Whitney. Pearson's or Spearman correlation analysis was used to evaluate the association between the inflammatory cytokines of IL-1β, the cartilage matrix of proteoglycan and collagen II and AQPs Mankin score.

## Results

### Cell viability, apoptosis and membrane permeability

**Fig 1:**
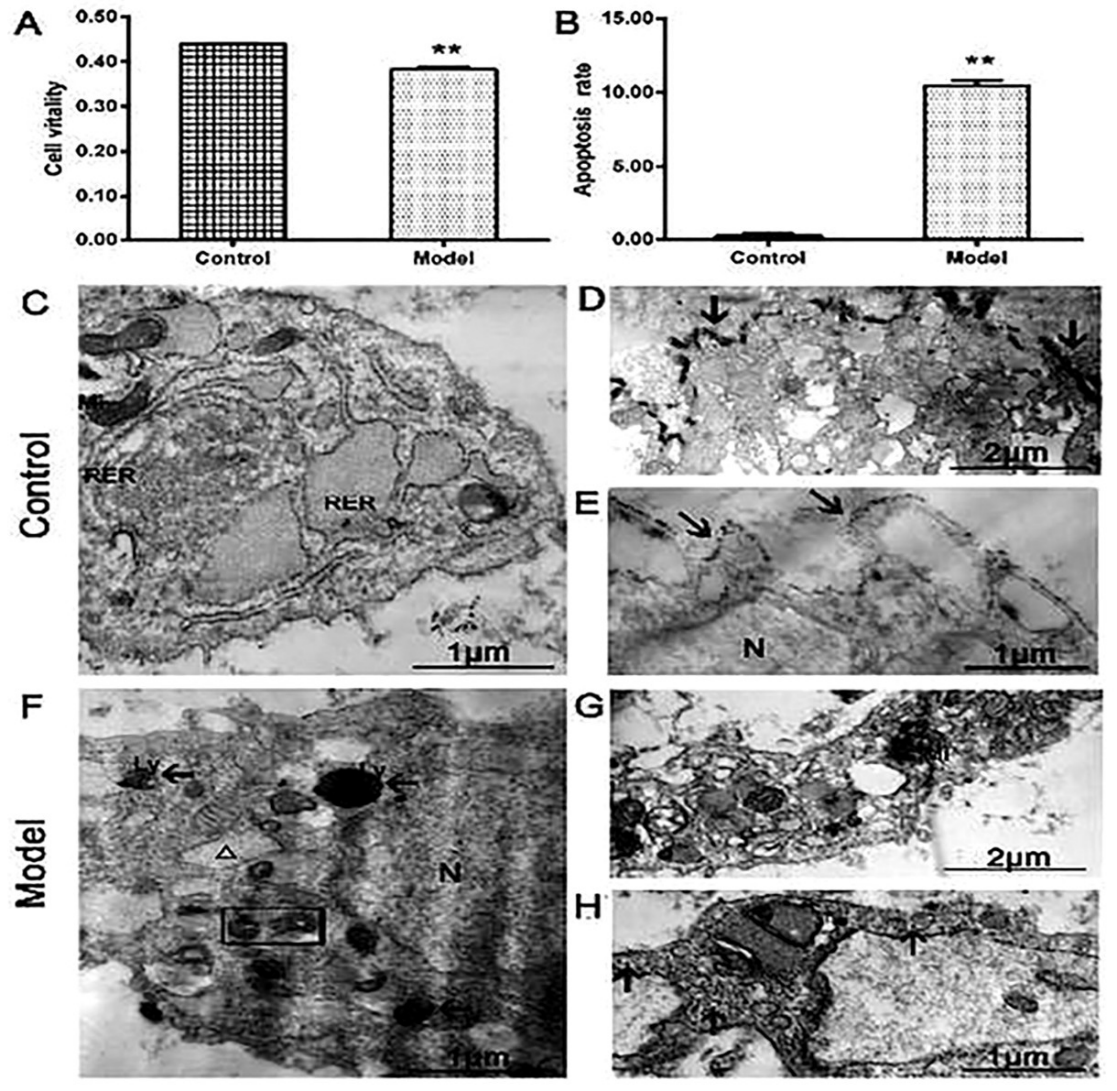
(A) The cell viability in the model group was significantly lower compared with the control group. (B) The apoptosis cells were much higher in model group than those in the control group (n = 3 per group, ^**^P < 0.01: the control group vs. the model group). (C) The normal chondrocytes had abundant mitochondria and rough endoplasmic reticulum. The enlargement version showed that lanthanum ions precipitated outside the cell membrane (arrows, D and E). (F) The model group showed that IL-1β-induced chondrocytes had vacuolization and mitochondrial pyknosis, the tracer lanthanum ions appeared in the organelles or lysosomes. The enlargement version showed that lanthanum ions scattered in organelle and cytoplasm (arrows, G and H). (N) Nucleus, (Mi) mitochondria, (RER) rough endoplasmic reticulum, (Ly) lysosome, (G) lanthanum ions (arrows), mitochondria dissolved (rectangle), vacuolized endoplasmic reticulum (△).

The cell viability in the IL-1β-treated group was significantly lower than that in the control group (Fig 1A, *P*<0.01). The rate of apoptosis cells was noticeably higher in IL-1β-treated group than that of the control group (Fig 1B, *P*<0.01).

The control group showed that the cells had clearly mitochondrial crest and rich rough endoplasmic reticulum (Fig 1C), and tracer lanthanum ions attached outside the cell membrane, which failed to enter the cells (Fig 1D and 1E). By contrast, the IL-1β-treated group showed the more pyknotic mitochondria, cell cavities with swollen, vacuolated endoplasmic reticulum, and the tracer lanthanum ions appeared in the organelles or lysosomes (Fig 1F). The cells showed the degenerated cytoplasm with scattered tracer lanthanum ions (Fig 1G and 1H). These results showed that IL-1β could reduce the cells viability, enhance the apoptosis cells, and impair the cell membrane and organelles.

### Determination of matrix content in chondrocytes

**Fig 2:**
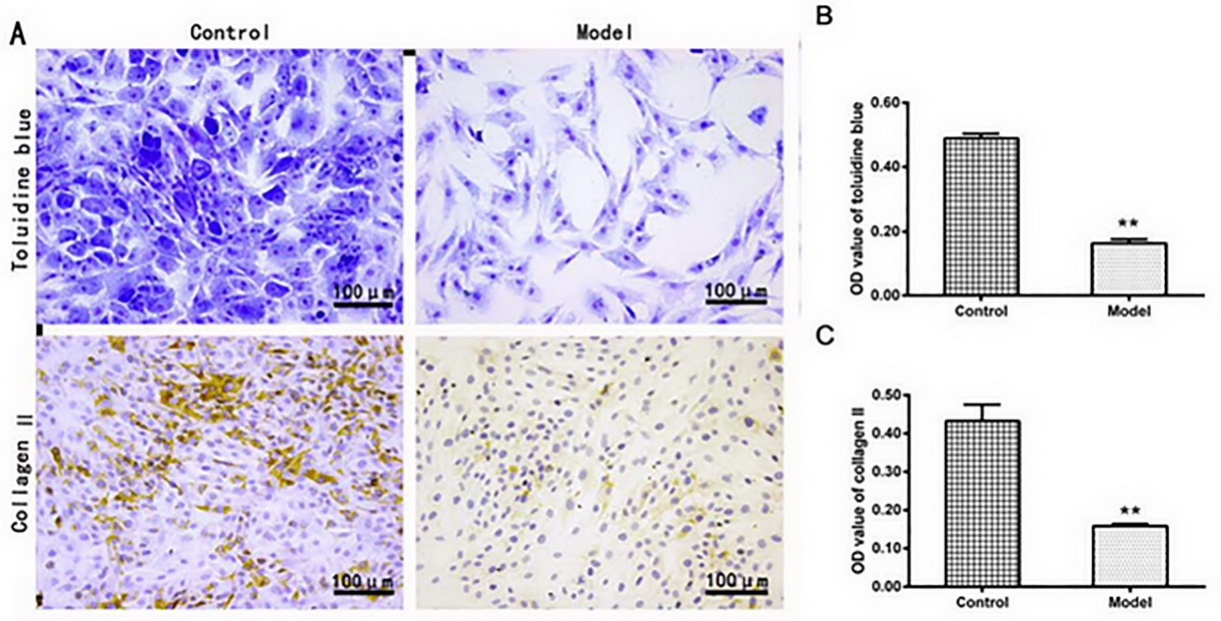
(A) The representative images of toluidine blue staining and collagen II immunohistochemical staining in chondrocytes, showing the proteoglycan or collagen II in the model group were significantly lower compared with the control group(B and C) as indicated by the semi-quantitative analysis (n = 3 per group. ^**^P < 0.01: the control group vs. the model group).

As showed in Fig 2A, the proteoglycan, the parts colored the blue-violet, was decreased in the IL-1β-treated group compared to the control group as indicated by the OD value (0.163 ± 0.013 vs. 0.489 ± 0.014, *P* <0.01, Fig 2B). The results suggested that the reduced proteoglycan would account for the reduction of proteoglycan-bound water in the IL-1β-induced chondrocytes. The fact that the water content distribution was similar to the proteoglycan contents, the loss of proteoglycan would lead to the decreased capability of water reserve in the IL-1β-induced chondrocytes.

Similar reduction of collagen II was found in immunocytochemistry experiments, the IL-1β-treated group showed the collagen II OD value (0.158 ± 0.006) was much lower than that in the control group (0.433 ± 0.043) (Fig 2C, *P* <0.01).

### Localization and quantitative analysis of AQPs in chondrocytes

**Fig 3:**
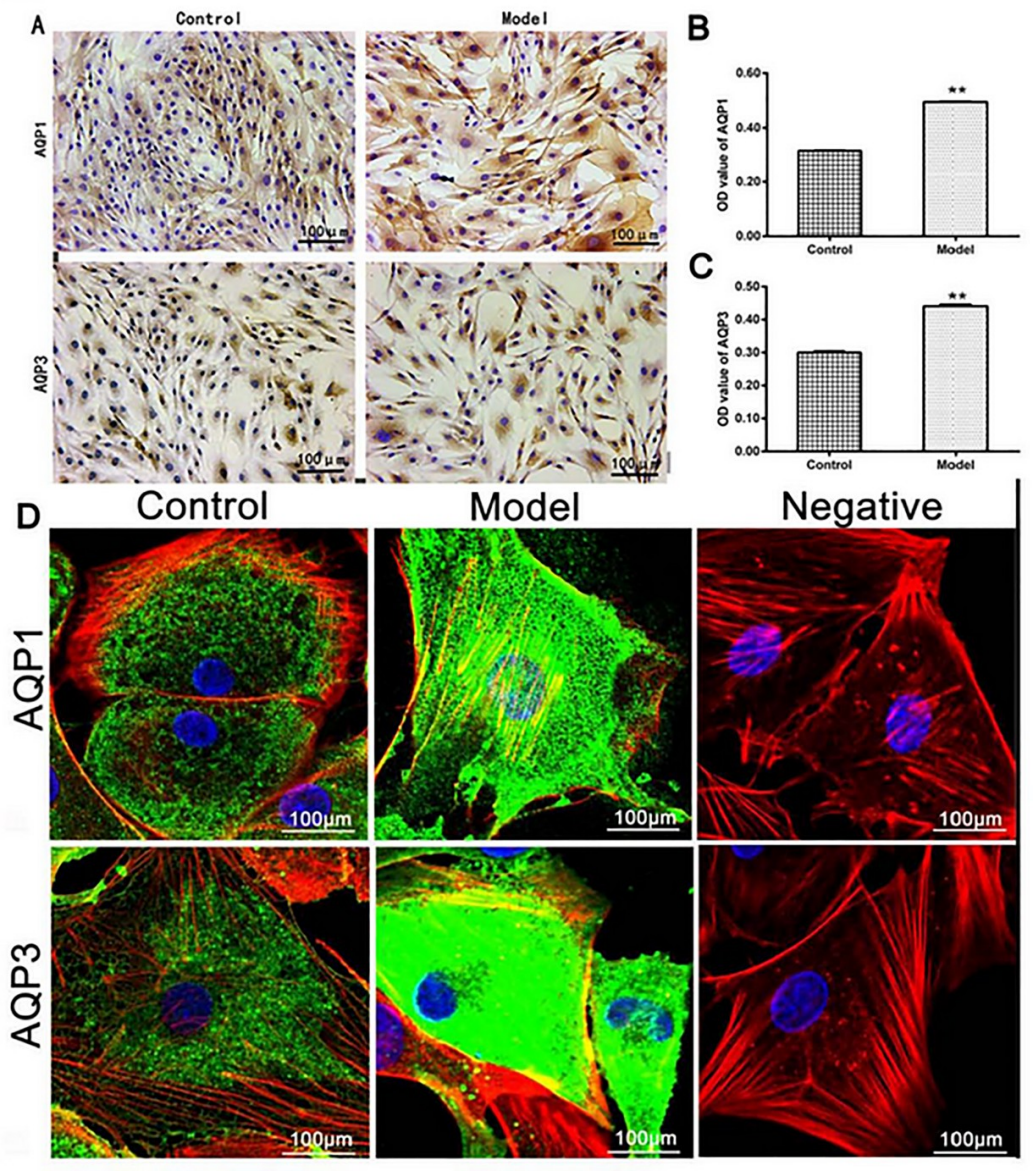
(A) AQP1 and AQP3 in the model group were significantly higher than those in the control group as indicated by semi-quantitative analysis (B and C). (D) The control group showed AQP1 and AQP3 (color green) evenly distributed in the cell membrane and cytoplasm, by contrast, AQP1 and AQP3 in the model group were increased in the plasma membrane and perinuclear cytoplasm or strongly expressed in the whole cell. The cell nuclei were stained with DAPI (colore blue), and the cytoskeleton were stained with rhodamine phalloidin (colore red). No expressions of AQPs were detected in the negative control group (n = 3 per group. ^**^P < 0.01 control group vs. model group).

As showed in Fig 3A, both AQP1 and AQP3 in the IL-1β-treated group were significantly higher than these in the control group, as indicated by protein OD value (*P* < 0.01, Fig 3B and 3C). In line with the AQPs protein expressions, the cells in the IL-1β-treated group exhibited the cytoplasmic edema with irregular nuclear shape (Fig 3A). These results indicated that IL-1β-induced higher expressions of AQP1 and AQP3 could increase the content of water in the chondrocytes.

Similar higher expressions of AQP1 and AQP3, as indicated by the green color in Fig 3D, were discovered in the IL-1β-treated group under the laser scanning confocal microscopy. In the IL-1β-treated group, both AQP1 and AQP3 were more pronounced in the cell membrane, cytoplasm or perinuclear, and the cells showed irregular cytomembrane, swelling cytoplasm and nucleus. Comparatively, little reactants were scattered in the cell membrane and cytoplasm, and cytomembrane, cytoplasm and nucleus showed regularly in the control group. No reactants of AQP1 and AQP3 were detected in the negative control.

### Histological changes of cartilage and matrix

**Fig 4:**
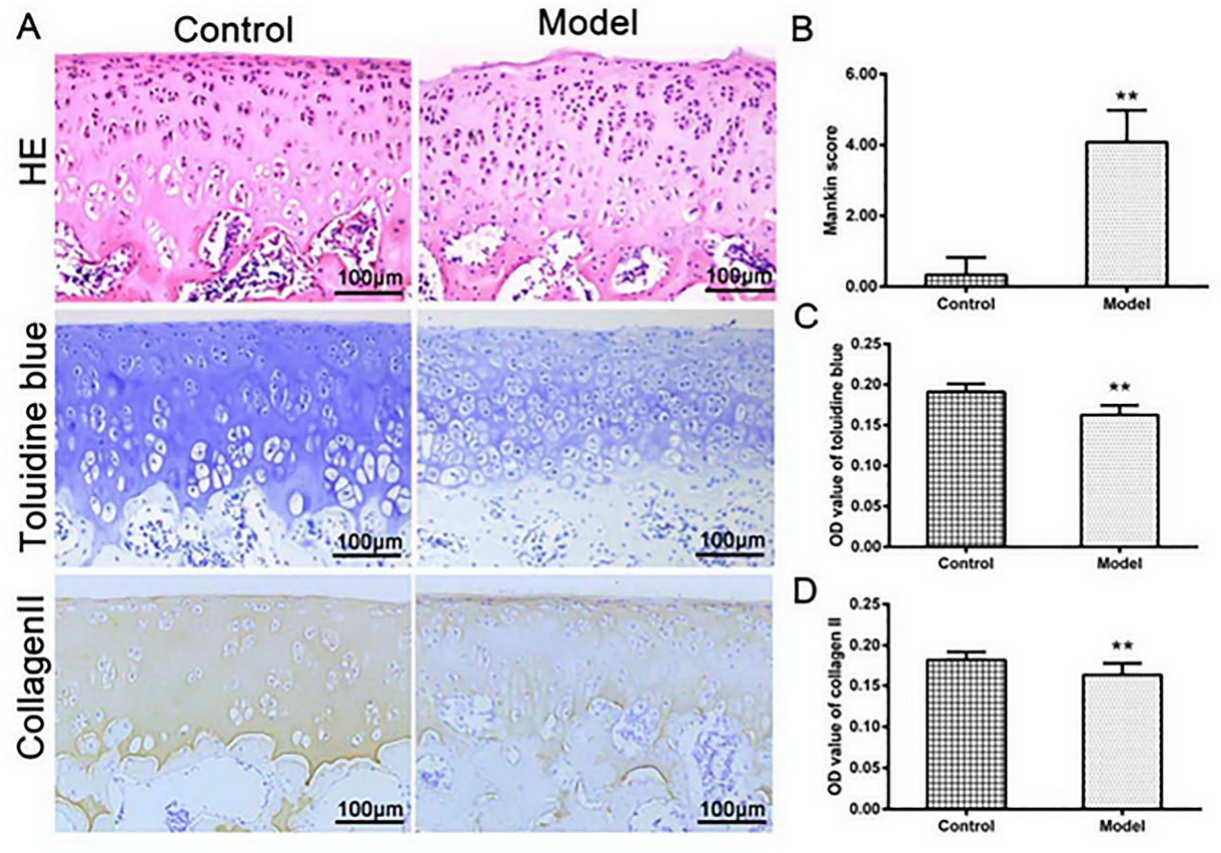
(A) HE stained showed a complete structure in normal cartilage, however, the model group showed the cartilage surface damaged with chondrocytes proliferated; Toluidine blue staining and collagen II showed a modest decrease in the model group compared with those in the control group, as indicated by the semi-quantitative analysis B, C and D (n = 12 per group, ^**^P < 0.01: the control group vs. the model group).

As showed in Fig 4A, the normal cartilage had a smooth surface with pleomorphic chondrocytes. The cells, with a spindle shape, were arranged densely with the long axis paralleled to the cartilage surface. In the middle layer, the chondrocytes were regular in shape with an oval nucleus; no aggregation of chondrocytes in the deep layer was detected. By contrast, the model group showed the cartilage surface damaged, and the chondrocytes in the middle layer clustered disorderly. According to Mankin score, the model group was the same as the early stage of OA (Fig 4B).

The control group exhibited the normal toluidine blue staining and collagen II staining, however, these staining results were significantly decreased in the model group (*P*<0.01, Fig 4A, 4C and 4D). These results suggested that the lost proteoglycan and collagen II were similar to the degeneration of early OA cartilage.

### Localization and quantitative analysis of AQPs in cartilage

**Fig 5:**
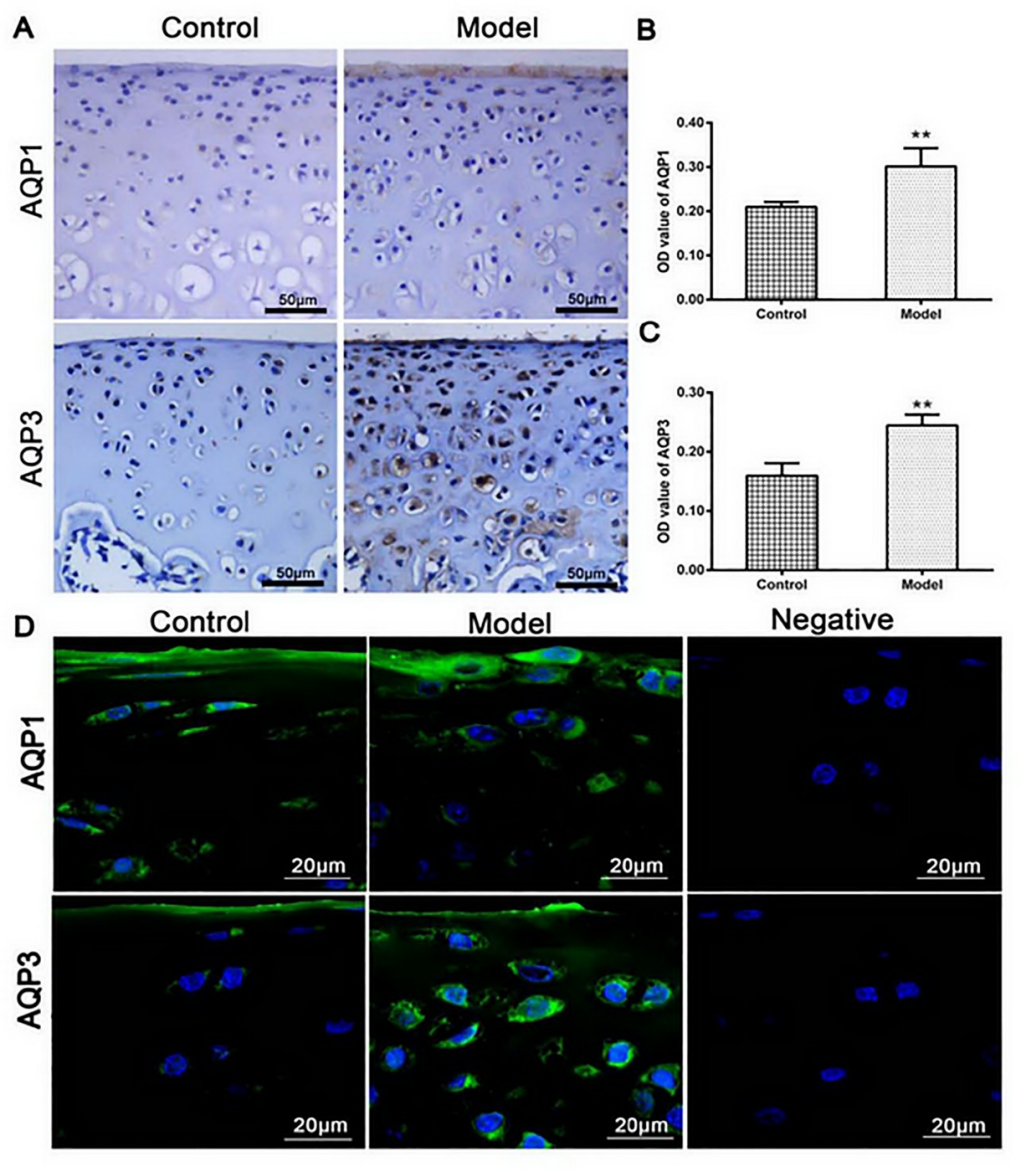
(A) The expressions of AQP1 and AQP2 in model group were higher than those in the control group as shown by the semi-quantitative analysis (B and C). Similar higher expressions and locations of AQPs were detected again by immunofluorescent assay (D, color green, the cell nuclei were stained with blue). Negative control was detected no expressions of AQPs (the control group vs. the model group ^**^P < 0.01, n = 12 per group).

In the cartilage, AQP1 was significantly higher on the surface, while AQP3 was mainly in the middle layer in the model group (Fig 5A), and the OD value of AQP1 and AQP3 in the model group was significantly higher than the control group (*P*<0.01, Fig 5B and 5C).

Similar higher levels and distributions of AQPs were detected again by immunofluorescent assay. As showed in the model group (Fig 5D), AQP1 was pronounced on the surface, while AQP3 was augmented in the middle layer, which was consistent with the results of immunohistochemistry. No expressions of AQP1 and AQP3 were detected in the negative control.

### IL-1β triggered the up-regulation of AQPs

**Fig 6:**
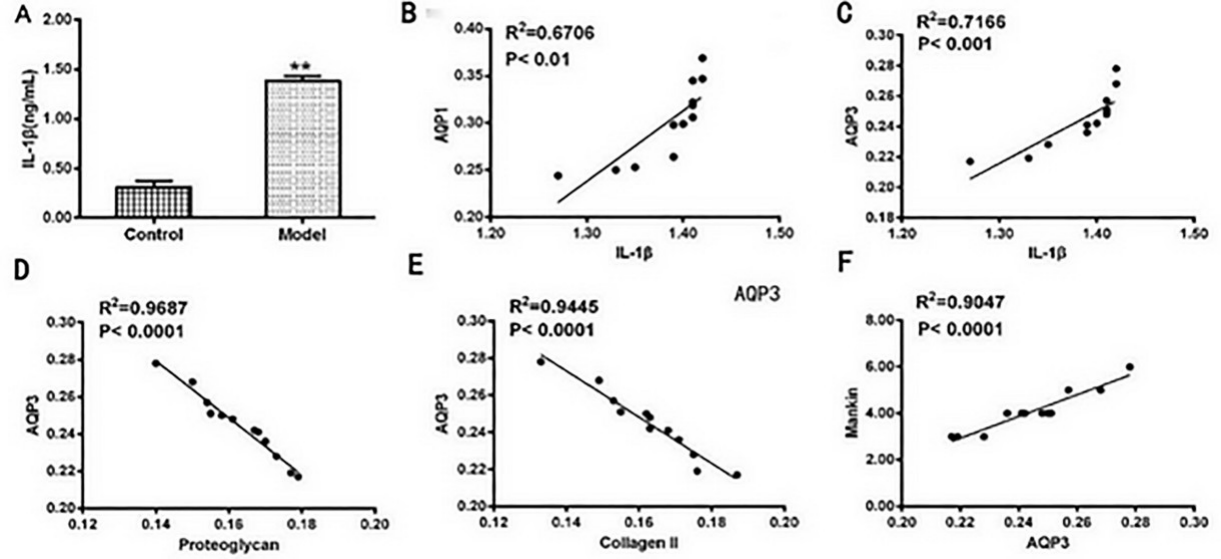
(A) IL-1β in the synovial fluid was significantly higher in the model group than that in the control group (^**^ *p* <0.01). Higher IL-1β was observed and correlated with AQP1 or AQP3 in the joint synovial fluid (B and C). The content of AQP3 (or AQP1, but not shown) was negatively correlated with cartilage matrix of proteoglycan (D) and collagen II (E) as indicated by the cartilage pathological Mankin’s score (F).

To further investigate the mechanisms of OA, the levels of IL-1β in the joint synovial fluid and AQPs in the joint cartilage were detected, as well as their correlations between the cartilage matrix were analyzed in the OA rat model group (Fig 6). As showed in Fig 6A, IL-1β in synovial fluid was significantly increased in the OA rat model group compared with that in the control group. Higher levels of IL-1β in synovial fluid were positively correlated with the levels of AQP1 (Fig 6B, R^2^ = 0.6706, *P*<0.01) and AQP3 (Fig 6C, R^2^ = 0.7166, *P*<0.001). The content of AQP3 (or AQP1, but not shown) was negatively correlated with the cartilage matrix of proteoglycan (Fig 6D) and collagen II (Fig 6E) as indicated by the cartilage pathological Mankin’s score (Fig 6F).

To further confirm that IL-1β triggered the up-regulation of AQPs, we injected IL-1β into the normal rat knee joints to observe the expressions of AQPs in the synovial fluid. Redness and swelling of joints, as well as similar high expressions of AQP1 and AQP3 in the synovial fluid were observed in the IL-1β-injected rat knee joints (results not shown).These data indicated that IL-1β via the up-regulation of AQP1 and AQP3, which reduced the cartilage matrix and accelerated progress of OA in the rat knee joints.

## Discussion

In our present study, we found that inflammatory cytokine IL-1β, via up-regulation of AQP1 and AQP3, promoted the abnormal water metabolism in the chondrocytes and resulted in the increase of free water and bound water (fluid) in the tissues that surround the joints. On the other hand, the massive loss of proteoglycans from the articular cartilage, in turn, led to an increase loss of bound water molecules and chondrocyte apoptosis, and ultimately accelerated the pathogenesis of OA.

Over 70% of the total tissue weight in the cartilage matrix consists of water [24], and the osteoarthritis cartilage in early-stage of OA is characterized by joint swelling with increased water content. Therefore, the abnormal water transport in the cartilage matrix and the metabolic water across the membranes of chondrocytes are involved in the pathological conditions of cartilage. [25, 26] As our experiment indicated, inflammatory cytokine IL-1β contributed to the pathogenesis of OA, this had to be conformity with the numerous research reports before.[27,28] However, anti-inflammatory therapy (nonsteroidal anti-inflammatory drugs, NSAIDs) clinically failed to control the pathogenesis of OA process, [29–33] as NSAIDs mainly inhibit cyclooxygenase to impair the production of prostaglandins, which are important mediators of both the pain and the inflammatory response. From our current experiments data indicated, AQPs may be as a potential target in the treatment of OA.

Aquaporins, as the integral membrane proteins, mainly serve as channels for the transfer of water across the membrane. So far, more than 10 isoforms have been discovered and expressed differentially in many types of cells and tissues. Our experiments found that AQP1 and AQP3 were mainly expressed in articular chondrocytes and up-regulated in the OA cartilage, which implicated in water- reserve and water balance disorders. The up-regulation of AQP1 and AQP3 accelerated the movement of water, which led to the joint swelling, increasing the tension within the collagen network.

In the early stage of OA, the joint swelling mechanism provided the extracellular matrix with the ability to resist tension and shear forces, thereby giving cartilage its ability to resist compression under a static or a dynamic mechanical load.[34] However, with the progression of OA, osteoarthritis cartilage showed extensive loss of proteoglycan, and its capability of water-reserve decreased. Early research found that AQP1 in human articular chondrocytes, synoviocytes and synovial microvessels was highly up-regulated in RA. AQP1 or other AQPs family members were involved in the movement of extracellular matrix and metabolic water across the membranes of chondrocytes and synoviocytes for the purposes of chondrocyte volume regulation and synovial homeostasis. [34]

On the other hand, high AQP1 and AQP3 mediated the acceleration of water transport could influence the ionic and osmotic conditions in cartilage, which in turn altered cell morphology and affected the viscoelastic mechanical properties of these cells, ultimately, affected the cells’ growth and metabolism. Recent research found that the up-regulated AQP1 in synovium and cartilage of rheumatoid arthritis patients was involved in joint swelling and synovial inflammation. Inhibition of AQP1 by acetazolamide showed a powerful therapeutic effect on rheumatoid arthritis. And this effect was via inhibiting NF-κB activation, suggesting AQP1 might be of potential target in rheumatoid arthritis treatment [35]. Besides, up-regulation of AQP1 and AQP3 were strongly correlated with chondrocyte apoptosis, part of the reasons that the abnormal water transport and the ionic and osmotic changes could alter the cellular environment and cell homeostasis. Also the upper-regulation of AQP1 could activate the activity of caspase-3 and result in chondrocytes apoptosis, which ultimately accelerated the development of osteoarthritis.[36] Early studies showed that the permeability of cell membrane mediated by AQPs can control the rate of apoptosis.[34]

Our experiments showed that AQP1 and AQP3 were more expressed in the cell membrane, cytoplasm or perinuclear (Fig 3D), or mainly in the proliferative zone and the upper mid-zone chondrocytes in the cartilage at the early stage of OA (Figs 4 and 5). Early research showed the expression of AQP3 (but not AQP1) was highly up-regulated with the progressing of the OA, and mainly located in the proliferative zone and the upper mid-zone chondrocytes throughout OA, [37] which was in line with our data. Status of AQPs distributions influenced the permeability of membrane organelles. [35] Higher expressions and different distributions of AQPs in the OA cartilage proved their involvement in the pathogenesis of OA, as articular chondrocytes are responsible for synthesizing and maintaining the extracellular matrix of the articular cartilage. [38]

In conclusion, inflammatory cytokines, via up-regulation of AQPs, accelerated the water metabolism and altered the ionic and osmotic conditions in the chondrocytes and resulted in the increase of free water and bound water in the tissues that surround the joints. Up-regulation of AQPs could cause the massive loss of proteoglycans from the articular cartilage, which exacerbated the loss of bound water molecules in chondrocytes. Therefore, AQPs may be a candidate therapeutic target for prevention and treatment of osteoarthritis.

## References

[1] Li X, Lang W, Ye H, Yu F, Li H, Chen J, et al (2013) Tougu Xiaotong capsule inhibits the tidemark replication and cartilage degradation of papain-induced osteoarthritis by the regulation of chondrocyte autophagy. Int J Mol Med 31:1349–1356. 10.3892/ijmm.2013.1341 23589102

[2] Liao N, Huang Y, Ye J, Chen W, Li ZF, Lin R, et al (2015) Protective effects of Tougu Xiaotong capsule on tumor necrosis factor-alpha-injured UMR-106 cells. Exp Ther Med 10:1908–1914. 10.3892/etm.2015.2739 26640571 PMC4665338

[3] Liess C, Lusse S, Karger N, Heller M, Gluer CC. (2002) Detection of changes in cartilage water content using MRI T2-mapping in vivo. Osteoarthritis Cartilage 10:907–913. 12464550 10.1053/joca.2002.0847

[4] Laurent D, Wasvary J, O'Byrne E, Rudin M. (2003) In vivo qualitative assessments of articular cartilage in the rabbit knee with high-resolution MRI at 3 T. Magn Reson Med 50:541–549. 10.1002/mrm.10566 12939762

[5] Verkman AS. (2009) Knock-out models reveal new aquaporin functions. Handb Exp Pharmacol 190:359–381. 10.1007/978-3-540-79885-9_18 PMC3595129 19096787

[6] Gao HF, Ren GL, Xu Y, Jin SZ, Jiang YQ, Lin JS, et al (2011) Research on the expressions of aquaporin-1 and chondrocytes apoptosis in osteoarthritis. Chin J prosthodontics Surg 3:279–284. 21500577

[7] Musumeci G, Leonardi R, Carnazza ML, Cardile V, Pichler K, Weinberg AM, et al (2013) Aquaporin 1 (AQP1) expression in experimentally induced osteoarthritic knee menisci: an in vivo and in vitro study. Tissue Cell 45:145–152. 10.1016/j.tice.2012.10.004 23164158

[8] Meng JH, Ma XC, Li ZM, Wu DC. (2007) Aquaporin-1 and aquaporin-3 expressions in the temporo-mandibular joint condylar cartilage after an experimentally induced osteoarthritis. Chin Med J (Engl) 120:2191–2194. 18167200

[9] Dijkgraaf LC, de Bont LG, Boering G, Liem RS. (1995) The structure, biochemistry, and metabolism of osteoarthritic cartilage: a review of the literature. J Oral Maxillofac Surg 53:1182–1192. 7562173 10.1016/0278-2391(95)90632-0

[10] Geyer M, Grassel S, Straub RH, Schett G, Dinser R, Grifka J, et al (2009) Differential transcriptome analysis of intraarticular lesional vs intact cartilage reveals new candidate genes in osteoarthritis pathophysiology. Osteoarthritis Cartilage 17:328–335. 10.1016/j.joca.2008.07.010 18775662

[11] Hagiwara K, Shinozaki T, Matsuzaki T, Takata K, Takagishi K. (2013) Immunolocalization of water channel aquaporins in human knee articular cartilage with intact and early degenerative regions. Med Mol Morphol 46:104–108. 10.1007/s00795-013-0014-3 23345027

[12] Chen Q, Zhang B, Yi T, Xia C.(2012) Increased apoptosis in human knee osteoarthritis cartilage related to the expression of protein kinase B and protein kinase Cá in chondrocytes. Folia Histochem Cytobiol 50:137–143. 10.2478/18709 22532149

[13] Zhou PH, Liu SQ, Peng H. (2008) The effect of hyaluronic acid on IL-1beta-induced chondrocyte apoptosis in a rat model of osteoarthritis. J Orthop Res;26:1643–1648. 10.1002/jor.20683 18524010

[14] Honorati MC, Cattini L, Facchini A. (2004) IL-17, IL-1beta and TNF-alpha stimulate VEGF production by dedifferentiated chondrocytes. Osteoarthritis Cartilage 12:683–691. 10.1016/j.joca.2004.05.009 15325633

[15] Shakibaei M, Allaway D, Nebrich S, Mobasheri A. (2012) Botanical Extracts from Rosehip (Rosa canina), Willow Bark (Salix alba), and Nettle Leaf (Urtica dioica) Suppress IL-1beta-Induced NF-kappaB Activation in Canine Articular Chondrocytes. Evid Based Complement Alternat Med 2012:509383 10.1155/2012/509383 22474508 PMC3312281

[16] Chowdhury TT, Bader DL, Lee DA. (2001) Dynamic compression inhibits the synthesis of nitric oxide and PGE(2) by IL-1beta-stimulated chondrocytes cultured in agarose constructs. Biochem Biophys Res Commun 285:1168–1174. 10.1006/bbrc.2001.5311 11478777

[17] Saklatvala J. (1986) Tumour necrosis factor alpha stimulates resorption and inhibits synthesis of proteoglycan in cartilage. Nature 322:547–549. 10.1038/322547a0 3736671 PMC7095107

[18] Shakibaei M, Schulze-Tanzil G, John T, Mobasheri A.(2005) Curcumin protects human chondrocytes from IL-l1beta-induced inhibition of collagen type II and beta1-integrin expression and activation of caspase-3: an immunomorphological study. Ann Anat 187:487–497. 16320828 10.1016/j.aanat.2005.06.007

[19] Li H, Li X, Liu G, Chen J, Weng X, Liu F, et al (2013) Bauhinia championi (Benth.) Benth. polysaccharides upregulate Wnt/beta-catenin signaling in chondrocytes. Int J Mol Med 32:1329–1336. 10.3892/ijmm.2013.1527 24129747

[20] Zhang XH, Xu XX, Xu T. (2015) Ginsenoside Ro suppresses interleukin-1beta-induced apoptosis and inflammation in rat chondrocytes by inhibiting NF-kappaB. Chin J Nat Med 13:283–289. 10.1016/S1875-5364(15)30015-7 25908625

[21] Zhao H, Zhang T, Xia C, Shi L, Wang S, Zheng X, et al (2014) Berberine ameliorates cartilage degeneration in interleukin-1beta-stimulated rat chondrocytes and in a rat model of osteoarthritis via Akt signalling. J Cell Mol Med 18:283–292. 10.1111/jcmm.12186 24286347 PMC3930415

[22] Murat N, Karadam B, Ozkal S, Karatosun V, Gidener S. (2007) Quantification of papain-induced rat osteoarthritis in relation to time with the Mankin score. Acta Orthop Traumatol Turc 41:233–237. 17876125

[23] Barton NJ, Stevens DA, Hughes JP, Rossi AG, Chessell IP, Reeve AJ, et al (2007) Demonstration of a novel technique to quantitatively assess inflammatory mediators and cells in rat knee joints. Journal of Inflammation 4:13 10.1186/1476-9255-4-13 17567894 PMC1919375

[24] Dijkgraaf LC, de Bont LG, Boering G, Liem RS. (1995) The structure, biochemistry, and metabolism of osteoarthritic cartilage: A review of the literature. J Oral Maxillofac Surg 53:1182–1192. 7562173 10.1016/0278-2391(95)90632-0

[25] Kraus VB, Blanco FJ, Englund M, Karsdal MA, Lohmander LS. (2015) Call for standardized definitions of osteoarthritis and risk stratification for clinical trials and clinical use. Osteoarthritis Cartilage 23:1233–1241. 10.1016/j.joca.2015.03.036 25865392 PMC4516635

[26] Bradley JD, Brandt KD, Katz BP, Kalasinski LA, Ryan SI. (1992) Treatment of knee osteoarthritis: relationship of clinical features of joint inflammation to the response to a nonsteroidal antiinflammatory drug or pure analgesic. J Rheumatol 19:1950–1954. 1294745

[27] Taruc-Uy RL, Lynch SA. (2013) Diagnosis and treatment of osteoarthritis. Prim Care 40:821–836. 10.1016/j.pop.2013.08.003 24209720

[28] Yang Y, Wang Y, Wang Y, Zhao M, Jia H. (2016) Tormentic Acid Inhibits IL-1β-Induced Inflammatory Response in Human Osteoarthritic Chondrocytes. Inflammation 39:1–9. 10.1007/s10753-015-0215-0 27102898

[29] Wang SN, Xie GP, Qin CH, Chen YR, Zhang KR, Li X, et al (2015) Aucubin prevents interleukin-1 beta induced inflammation and cartilage matrix degradation via inhibition of NF-κB signaling pathway in rat articular chondrocytes. Int Immunopharmacol 24:408–415. 10.1016/j.intimp.2014.12.029 25576403

[30] Permuy M, Guede D, Lopez-Pena M, Munoz F, Caeiro JR, Gonzalez-Cantalapiedra A. (2015). Effects of diacerein on cartilage and subchondral bone in early stages of osteoarthritis in a rabbit model. BMC Vet Res 11:143 10.1186/s12917-015-0458-x 26135886 PMC4487570

[31] Minguzzi M, Cetrullo S, D'Adamo S, Silvestri Y, Flamigni F, Borzi RM. (2018) Emerging Players at the Intersection of Chondrocyte Loss of Maturational Arrest, Oxidative Stress, Senescence and Low-Grade Inflammation in Osteoarthritis. Oxid Med Cell Longev 2018:3075293 10.1155/2018/3075293 29599894 PMC5828476

[32] Huang G, Hua S, Yang T, Ma J, Yu W, Chen X. (2018) Platelet-rich plasma shows beneficial effects for patients with knee osteoarthritis by suppressing inflammatory factors. Exp Ther Med 15:3096–3102. 10.3892/etm.2018.5794 29599843 PMC5867452

[33] Raman S, FitzGerald U, Murphy JM. (2018) Interplay of Inflammatory Mediators with Epigenetics and Cartilage Modifications in Osteoarthritis. Front Bioeng Biotechnol 6:22 10.3389/fbioe.2018.00022 29594113 PMC5861204

[34] Trujillo E, Gonzalez T, Marin R, Martin-Vasallo P, Marples D, Mobasheri A. (2004) Human articular chondrocytes, synoviocytes and synovial microvessels express aquaporin water channels; upregulation of AQP1 in rheumatoid arthritis. Histol Histopathol 19: 435–444. 10.14670/HH-19.435 15024704

[35] Cai L, Chen WN, Li R, Hu CM, Lei C, Li CM. (2018) Therapeutic effect of acetazolamide, an aquaporin 1 inhibitor, on adjuvant-induced arthritis in rats by inhibiting NF-\B signal pathway. Immunopharmacol Immunotoxicol 40:117–125 10.1080/08923973.2017.1417998 29303021

[36] Gao H, Gui J, Wang L, Xu Y, Jiang Y, Xiong MY, et al (2016) Aquaporin 1 contributes to chondrocyte apoptosis in a rat model of osteoarthritis. Int J Mol Med 38:1752–1758. 10.3892/ijmm.2016.2785 27779640 PMC5117737

[37] Jh Meng, Ma XC, Li ZM, Wu DC. (2007) Aquaporin-1 and aquaporin-3 expressions in the temporo-mandibular joint condylar cartilage after an experimentally induced osteoarthritis.Chin Med J 120:2191–2194 18167200

[38] Cohen NP, Foster RJ, Mow VC. (1998) Comlocalization and dynamics of articular cartilage: structure, function, and maintaining healthy state. J Orthop Sports Phys Ther 28: 203–215. 10.2519/jospt.1998.28.4.203 9785256

